# The Behavioral and Social Dimension of the Public Health System of European Countries: Descriptive, Canonical, and Factor Analysis

**DOI:** 10.3390/ijerph20054419

**Published:** 2023-03-01

**Authors:** Tetiana Vasylieva, Beata Gavurova, Tetiana Dotsenko, Svitlana Bilan, Marcin Strzelec, Samer Khouri

**Affiliations:** 1 The London Academy of Science and Business, London W1U 6TU, UK; 2 Department of Financial Technologies and Entrepreneurship, Sumy State University, 40000 Sumy, Ukraine; 3 Faculty of Mining, Ecology, Process Control and Geotechnologies, Technical University of Kosice, 040 01 Kosice, Slovakia; 4 Economic Cybernetics Department, Sumy State University, 40000 Sumy, Ukraine; 5 Institute of Sociology, Technical University of Berlin, 10623 Berlin, Germany; 6 Department of Humanities and Social Sciences, Rzeszow University of Technology, 35-959 Rzeszow, Poland; 7 Academy of Justice, 02-520 Warsaw, Poland

**Keywords:** healthcare system, level of healthcare system development, behavioral aspect, social aspect, integral indicator, descriptive analysis, cluster analysis, canonical analysis, factor analysis

## Abstract

Background: The state and prospects of the healthcare industry of a country are among its top priorities because the quality of life and health of its citizens are indicators of its success and competitiveness. The aim of this study is to conduct a theoretical analysis and qualitative and quantitative assessments of indicators by developing an integral indicator in the context of behavioral, social, demographic, and economic factors that characterize the level of healthcare system development in European countries using multivariate statistical modeling methods. Methods: The study was implemented using Statistica 10 and Statistica Portable statistical packages. The statistical base of the study was formed using descriptive analysis; a group of 10 European countries was identified using a cluster analysis based on the application of an iterative divisive k-means method. The degree and significance of the interrelations between the components characterizing the studied groups of indicators were determined using canonical correlations by conducting a canonical analysis. Factor modeling is conducted by applying the analysis of the main components to determine the relevant indicators for assessing the level of healthcare system development to build integral indicators of the level of healthcare system development in European countries. Results: The need to improve the level of healthcare system development in European countries was confirmed. Shortcomings and possible reserves for potential improvement of the healthcare system were identified. Conclusions: The results can help public authorities, officials and employees of the healthcare sector organize and conduct effective, timely, high-quality regulation and adjustment of the regulatory and legislative framework to improve healthcare system development.

## 1. Introduction

Currently, the state and prospects of the healthcare industry are among the top-priority issues in European countries. Socially oriented states involve the parallel development of the economic, political, and modern technological sectors, as well as the efficiency and quality of the medical sector, to improve the population’s quality of life. The quality of life and health of its people constitute an indicator of the success and competitiveness of a country. Therefore, the attention of states and societies is focused on the sustainable development of the medical system to make it able to respond flexibly and adapt to new complex and large-scale challenges.

In recent years, there has been significant progress in the health systems of European countries. Ensuring the proper quality of medical care is one of the main tasks of the transformation processes of European states. However, some states have many questions about the medical industry. This requires strengthening national health systems and developing policies and strategies for medical systems at the level of certain countries.

In response to modern challenges related to human health threats, relevant officials are constantly monitoring, evaluating, and developing progressive health policies in all European countries. These policies will allow for adapting medical services to the population’s needs, identifying the latest priorities in the rapidly changing and difficult financial and economic situation, choosing the best medical technologies, and regulating the balance between therapeutic and preventive measures. Health authorities strive to improve the efficiency of the health system to improve people’s health and meet the growing expectations of public satisfaction with medical services. 

In turn, improving and regulating the medical system and the quality of medical services requires understanding the indicators used to measure and evaluate the healthcare system. Currently, there are many qualitative and quantitative indicators for measuring various health system characteristics used in countries worldwide. Such key indicators for each country may differ significantly, depending on the socio-economic and political development of the country. Therefore, it is essential to define the most relevant indicators of each country and why it is necessary to introduce and ensure international world standards of the healthcare system to be able to compare and analyze the experiences of advanced countries around the world in this matter in the future.

It should be noted that health scientists within countries identify certain official sets of indicators with different balances of the key aspects used by the state regulatory authorities in the medical fields of their specifc country. The following scientists have studied the development, research, analysis, and monitoring of the dimension indicators of the health care system: Beaussier A. et al. [1] highlighted indicators of health care quality measurement; Khan S. et al. [2] explored public health indicators; Gartner J. et al. [3] reviewed key health performance indicators; Labella B. et al. [4] calculate patient safety indicators; and Carini E. et al. [5] evaluated the performance indicators of medical institutions, etc. Other notable publications include Lyeonov S. et al. [6], Tiutiunyk I. V. et al. [7], Smiianov V. A. et al. [8], Aliyeva Z. [9], Kolosok S. et al. [10], Piven D. et al. [11], Shipko A. et al. [12], Strangfeldova J. et al. [13], Privara A. [14], and Ivankova V. et al. [15].

The peculiarities of the formation of the integral health care system indicators are reflected in the scientific studies of the following scientists: Stelzer D. et al. [16] suggest the use a regional integrated health care model “Healthy Kinzigtal” using quality indicators for optimizing health care and economic efficiency, and van den Akker E. F. M. M. et al. [17] describe the development and implementation of a comprehensive integral assessment approach to health status in patients, etc. Particular attention should be paid to the paper by Vasilyeva T. et al. [18] on the formation of integral indicators of the socio-political and economic situation in a country for the assessment of the dynamics of bifurcation transformations in the economy. Cabinova V. et al. [19] is another noteworthy source on the topic.

It is also worth researching the degree of satisfaction with medical services. This issue is quite specific and has mixed coverage in the scientific literature. However, the following specialists are engaged in particular approaches to this topic: Baranska A. et al. [20] conducted an assessment of the level of satisfaction with medical care among patients as an indicator of the quality of medical care; Vitale E. et al. [21] investigated the satisfaction level of the population regarding health care during the COVID-19 pandemic; Man E. et al. [22] defined patient satisfaction with private recovery services during the COVID-19 pandemic; Ren L. et al. [23] presented a survey on the cross-sectional degree of consumer community satisfaction with the primary care system; Lee Y. et al. [24] studied the subjective frame of patient satisfaction with the comprehensive nursing service; Smith et al. [25] tested the Determinants of Hospital Service Quality; and Carmo Caccia-Bava M. et al. [26] revealed important factors for success in hospitals. Other notable publications on this topic include Probst D.T. et al. [27], Louis R. [28], Lesniewski, M.A. [29], Mrabet S. et al. [30], Gavurova B. et al. [31], Zaharia R. et al. [32], Gavurova B. et al. [33]., Halicka K. et al. [34], Rosenberg D. et al. [35], and Zain N.A.M. et al. [36].

Studying the scientific achievements of scientists regarding the research methods used in world practice, we note that factor analysis is one of the most common methods used in scientific research, as evidenced by information from the Scopus database, 3,664,361 publications on the topic. Moreover, factor analysis is most often used by specialists in the field of medicine, and publications in this field account for 49.06% of the total volume of published papers indexed in Scopus related to factor analysis. Using this research method, healthcare scientists have solved some complex analytical problems, such as machine methods of risk factor analysis and prediction of epidemiological studies proposed by Tran V. et al. [37]; a factor analysis study on the public’s perspectives of qualities and behaviors a good doctor by Grundnig J. S. et al. [38]; and many others. Zhang L. et al. [39], Kuzior A. et al. [40], Rajan D. [41], Awojobi O.N. [42], Hinrichs G. et al. [43], and Kadar B. et al. [44] are other notable works employing factor analysis. Social sciences researchers also use the factor analysis method in their work (6.48%). The paper by Vasilyeva T. et al. [45,46,47], regarding the use of factor analysis as one of the methods in modeling social and economic patterns associated with the COVID-19 pandemic, arouses particular interest. Researchers from other scientific fields also widely use factor analysis in their works (44.46%). Considerable attention should be paid to the works of the following scientists using the elements of factor analysis: Kuzmenko O. V. et al. [48], regarding an approach to managing innovation to protect the financial sector against cybercrime, and Kuzmenko O. V. et al. [49] who employed factor analysis in the economic modeling to determine the influence of relevant indicators of gender policy on the efficiency of a banking system. Other notable papers in this regard include Didenko, I. et al. [50]; Brychko M. et al. [51]; Dao L.T. et al. [52]; Streimikiene D. [53]; Coman I. et al. [54]; Uslu A. et al. [55]; Ibe R. et al. [56]; and Quinonez Tapia F. et al. [57].

According to the Scopus database, scientists widely use descriptive analysis in many branches of science (186,060 publications). Descriptive analysis is most commonly used in health system research—60.90% of the total number of publications related to descriptive analysis indexed in Scopus were from health system research (with 49.41% in the field of medical knowledge 11.49% in nursing). Thus, modern healthcare scientists suggest studying various issues using the descriptive analysis method. For example, Riediger N. D. et al. [58] used descriptive analysis of food pantries in twelve American states; Taljaard L. et al. [59] conducted a descriptive analysis of the case mix in East London, South Africa; Bayou N. B. et al. [60] used a descriptive structural analysis of quality of labor and delivery care in Ethiopia; and Wylie C. A. et al. [61] described a retrospective descriptive analysis. Descriptive analysis is also very often used in social sciences (18.56%). Scientists in this field are currently studying some aspects based on descriptive analysis, which include a descriptive analysis of complaints related to COVID-19 in California by Thomas M. D. et al. [62]; a descriptive qualitative analysis of older adults’ accounts in Chile by Shura R. et al. [63]; and others such as Njegovanovic A. [64], Sarihasan I. et al. [65], Pop R.-A. et al. [66], Dai X. et al. [67], Xuechang Zhu et al. [68], Halina Waniak-Michalak et al. [69], and Zaharia R. M. et al. [70]. Other scientific fields account for 20.54% of scientific publications in the Scopus database related to descriptive analysis. Lyeonov S. et al. [71] used descriptive analysis during the implementation of gravitational and intellectual data analysis to assess the money laundering risk of financial institutions, as well as Tommaso F. D. [72], Gallo P. et al. [73], Barrientos-Baez A. et al. [74], Alshoubaki H. et al. [75], Vysochan O. et al. [76], Belascu L. et al. [77], Kramarova K., Svabova L. et al. [78], Wildowicz-Szumarska A. [79], Blazevic Bognar Z. et al. [80], Loi N. T. N. [81], and Capolupo N. et al. [82].

The use of the canonical analysis toolkit is three times less than that of descriptive analysis, but it still attracts considerable interest among scientists (57,910 publications, according to the Scopus database). Most often, biochemistry, genetics, and molecular biology specialists use canonical analysis in their research (27.05% of the total number of publications related to canonical analysis indexed in Scopus). In contrast to descriptive analysis, canonical analysis is less used in Medicine (13.85%) and Social Sciences (5.32%). The following works using canonical analysis deserve attention: a new insight from canonical correlation analysis when determining the relationship between the quality of work of medical professionals was presented by Wang W. et al. [83]; canonical correlation analysis of factors that influence the quality of life among patients was carried out by Liu Y. et al. [84]; the combination of canonical correlation analysis and holo-Hilbert spectral analysis was used by Lee P. et al. [85]; a canonical correlation analysis for testing the specificity of environmental risk factors for development was used by Bignardi G. et al. [86]; and canonical correlation analysis was applied in determining relationships between anthropometric variables by Malakar B. et al. [87]. Other notable uses of canonical analysis include Lyeonov S. V. et al. [88] and Gavurova B. et al. [89].

The aim of this study is to conduct a theoretical analysis and qualitative and quantitative assessments of indicators by developing an integral indicator in the context of behavioral, social, demographic, and economic factors that characterize the level of healthcare system development in European countries using multivariate statistical modeling methods.

Thus, the theoretical basis of the main constructions of the research model is based on well-known and proven methods of this branch of science, namely, cluster analysis [37,38,39,40,41,42,43,44,45,46,47,48,49,50,51,52,53,54,55,56,57], factor analysis [58,59,60,61,62,63,64,65,66,67,68,69,70,71,72,73,74,75,76,77,78,79,80,81,82], descriptive analysis, and canonical analysis [83,84,85,86,87,88,89]. It is also based on previous authors’ research [46,47,90], although these sources have not yet been properly disseminated in the field of health protection and have not been applied in the proposed integrated form of modeling.

Considering the existing threats to human health and, accordingly, the associated problems in the organization and functioning of health systems, this study will address such gaps in the knowledge of the health system as: imperfections and gaps in the complex assessment of the level of healthcare system development in terms of some important behavioral, social, demographic, and economic factors not being used in the work of specialists in this industry while characterizing the level of healthcare system development in European countries; imperfections in the existing integrated indicators of the health system, which do not take into account many of the relevant factors; the lack of effective methods and approaches for assessing the level of health systems in European countries.

## 2. Materials and Methods

The study was implemented in three stages based on the World Bank indicators for European countries from 2000 to 2020 using Statistica 10 and Statistica Portable statistical packages. 

When conducting the study, the following restrictions were imposed for the study countries: in the first stage of the study, 42 European countries were selected from all countries of the world, 10 of which were studied in the second and third stages, selected by cluster analysis; for the study period, years from 2000 to 2020 were used.

Stage 1 of the study included the formation and modeling of the statistical base of the study (input data array) using descriptive analysis and applying the Statistica 10 and Statistica Portable statistical packages.

To build models that would help establish the influence of certain factors on the indicator of the level of healthcare system development in European countries, it was proposed to define four groups of indicators according to the following constituent features: behavioral, social, demographic, and economic. The information base of the study is based on the World Bank indicators, which determine the public health system’s behavioral, social, and demographic features in conjunction with the economic aspect.

The source of the research data is the World Bank database—a reliable database of statistical data created by specialists at the World Bank. The formation and dissemination of this database are based on internationally accepted professional standards. The World Bank also cooperates with the international scientific and statistical community, namely, UN agencies, the Organization for Economic Cooperation and Development, the International Monetary Fund, and regional development banks. The World Bank ensures that all data in its database are high-quality and complete.

The selection of European countries for the study was based on cluster analysis [91], which was conducted using Statistica 10 and Statistica Portable. The clustering of countries was based on iterative divisional k-means clustering. Four key indicators were initially selected as a statistical base for the further clustering of countries. Then, cluster analysis verified the adequacy of dividing 42 European countries into groups. To evaluate and compare clusters, we used variance analysis to select clusters 3, 4, and 5. At the descriptive analysis stage (including cluster analysis) of throughput indicators, the values of the intergroup variances (between SS) and intragroup variances (within SS) of these characteristics, the value of the Fisher criterion (F), and the probability of a possible rejection of the null hypothesis (p) were determined to indicate the adequacy of clustering. 

We used the the “Multivariate Exploratory Techniques”—“Canonical Analysis” package of the Statistica toolkit at this stage, where k means clustering (k-means method)—i.e., the analysis of variation (variance analysis)—was used to directly identify specific groups of countries that are typical of their characteristics.

Stage 2 of the study involved the determination of the degree and significance of the interrelations between the components characterizing indicators (behavioral, social, demographic, economic) selected for the integral indicator’s construction of the level of healthcare system development in European countries using canonical analysis. This stage involves causal analysis, i.e., identifying which groups of indicators are the causes of the issue under study and which are the consequences. Thus, canonical analysis makes it possible to reduce a multidimensional set of characteristic features to a narrower concentrated system consisting of pairs of elements that are the most correlated with each other. This procedure allows for a statistical assessment of the significance and relationships of the studied features.

Mathematically, the primary purpose of canonical analysis involves determining the correlation of weighted sums, linear combinations called “canonical variables”, from each possible set of characteristics that make up causal and effective features. The procedure for implementing canonical analysis uses the Statistica 10 and Statistica Portable packages. This approach makes it possible to obtain a processed standardized source base of easy-to-analyze tabular data (Table 1). 

In the above Table, x≥y, x is the number of system attributes in the first equation from the first group, and y is the number of system attributes in the first equation from the second group. 

The estimation of the relationship between canonical variables H and F is described in Formula (1):

(1) $$\{\begin{array}{c} H=p_{1}a_{1}+p_{2}a_{2}+\ldots +p_{x}a_{x}\\ F=q_{1}b_{1}+q_{2}b_{2}+\ldots +q_{y}b_{y} \end{array}$$

where $p_{i},\;\left(i=\bar{1,\;x}\right)$, $q_{j},\;\left(j=\bar{1,\;y}\right)$ are the corresponding weights of coefficients calculated when solving a problem with eigenvalues. Moreover, the change in these weights determines the difference in the value of canonical variables and the canonical correlation coefficient k (Formula (2)). In turn, the canonical correlation coefficient evaluates the dependence of two variables and determines the density of the dependence between canonical variables:

(2) $$k=\frac{\mathrm{cov}\left(H,F\right)}{\sqrt{\mathrm{var}\left(H\right)\mathrm{var}\left(F\right)}}$$

The significance of correlation dependence is assessed using a standard statistical criterion (significance level—probability of error) υ and the standard statistical confidence interval. Moreover, a higher significance level value corresponds to a lower level of trust.

Statistica 10 and Statistica Portable software tools were used to implement canonical analysis. Initially, panel data were generated as a table of input data for the period for 10 European countries selected according to cluster analysis. Next, the dependence between groups of indicators was alternately determined in six tables by applying the function “Multivariate Exploratory Techniques”—“Canonical Analysis”.

Based on the generated data, we first checked the adequacy of the analysis. At this stage of the canonical analysis, the bandwidth indicators are as follows: R is the canonical correlation coefficient showing the strength and direction of the relationship between groups of indicators (the R-value tends to 1); ${\mathrm{Chi}}^{2}$ is the adequacy criterion (the value of ${\mathrm{Chi}}^{2}$ tends to Infinity); P is the probability of rejecting the hypothesis that there is no relationship between groups of indicators (the *p*-value tends to 0); and the total redundancy, which shows how much the variation of one group is explained by the variation of another group (from 0% to 29%—no connection; from 30% to 49%—a weak connection; from 50% to 69%—an average connection; from 70% to 100%—a strong connection) and shows which group of indicators’ % total redundancy is greater, where the group of indicators is a consequence.

Stage 3 of the study involves determining relevant indicators for assessing the level of healthcare system development in European countries (i.e., the significance of factors within each group of indicators to evaluate the results of the input array of analysis data), as well as the construction of an integral indicator of the level of healthcare system development in European countries. 

Factor analysis was performed using the principal component method. 

The selected type of factor analysis, i.e., principal components, is a mathematically based methodology that identifies relevant indicators and their structures, determines hidden indicators, establishes statistical relationships, and excludes non-influential indicators to simplify the analysis results. Factor analysis involves a simple logical construction of a generalization of the values of certain features and replaces correlated measures with uncorrelated factors. According to the method of principal components, the main components and generalized features are distinguished from the input indicators. Moreover, the mathematical model of the principal components method implies a logical assumption that a certain general result is produced from a set of interrelated features. Modeling according to this method provides the following [92] (Formula (3)):

- The beginning of the construction of the matrix of input indicators (R);
- Formation of a matrix of standardized values (S);
- Formation of the matrix of pair correlations (K);
- Formation of a diagonal matrix of eigenvalues (D), a matrix of unnormalized vectors (N), matrices of normalized vectors (V), and calculation of the contribution of variable indicators;
- The formed matrix of the factor expression (F) and the matrix of principal components (Q), as well as the values of the factors that allow determining the relevant factors and their weighting coefficients. Based on the weighting coefficients of the relevant factors, the weighted influence of the indicators under consideration is determined:
  (3) $$R\to S\to K\to \left\{\frac{D}{N\to V}\right\}\to F\to Q$$

Therefore, the method of principal components involves constructing a factor space in which variables and observations are simultaneously classified to form the principal components. A vector space of variables and observations is constructed. A matrix of correlations or covariances is formed to obtain a new system of uncorrelated variables, that is, principal components. Principal components are formed as linear combinations of initial variables.

That is, the input variables are transformed (the matrix of input indicators, R) into new variables (the matrix of principal components, Q).

Factor analysis is implemented using the Statistica 10 and Statistica Portable software packages, using the “Multivariate Exploratory Techniques” function “Principal Components and Classification Analysis”. The factor analysis was based on input data sampling, where panel data were taken as input data in the form of a table of initial data for the period from 2000 to 2020 for 10 European countries selected according to cluster analysis (Austria, Belgium, Germany, Denmark, Finland, France, the United Kingdom, the Netherlands, Norway, and Sweden). 

Within the framework of this stage, the principal components method was used to substantiate the expediency of considering the most influential ones that have the greatest weight in the group when assessing the level of development of the healthcare system in European countries.

All four social, demographic, economic, and behavioral groups of selected indicators were used for further analysis at this stage. However, according to the results of the canonical analysis, special attention is paid to groups of social and demographic indicators that significantly impact other groups of indicators.

To assess the level of healthcare system development in European countries, based on the data of indicators, a scree plot of eigenvalues of the correlation matrix of the input data was built using the “Multivariate Exploratory Techniques”, “Principal Components and Classification Analysis”, and “Scree plot” functions. “Multivariate Exploratory Techniques”, “Principal Components and Classification Analysis”, and “Eigenvalues” functions were used to form a table of eigenvalues of the correlation matrix and to derive statistical indicators of the group to assess the level of healthcare system development in European countries.

Conducting the eigenvalues analysis of the correlation matrix of the input data indicators for assessing the level of healthcare system development of European countries confirms that the first three factors determine the relevant indicators because, together, they account for at least 70% of the variation of the resulting characteristic. Then, using the “Multivariate Exploratory Techniques”, “Principal Components and Classification Analysis”, and “Contributions” functions, we attain variable indicators of the group to assess the level of healthcare system development in European countries.

The Table named “Intermediate values for the calculation of the relevance of indicators of the group for assessing the level of development of the healthcare system of European countries” was formed based on the eigenvalues of the correlation matrix of the input data to assess the level of healthcare system development of European countries considering the first three factors and the contribution of the variables. The information in this Table depicts the logic of calculating the arithmetic mean of the weighted impact of social indicators for assessing the level of healthcare system development in European countries on the value of this level by calculating the sum of the products of the weight coefficients of the factors, i.e., the eigenvalues of the correlation matrix of the input base of the selected factors. As a result of calculations, the values of the column “weighted impact of indicators” in Table 2 are obtained. That is, a weighted impact of indicators is obtained at the stage of the factor analysis of throughput indicators.

To define an integral indicator of the level of healthcare system development of European countries, we emphasize that a qualitative description of the structure of the studied healthcare system in terms of determining the level of healthcare system development of European countries requires the development of an integral indicator, either using all the main components or a large enough quantity for analysis. Thus, we use the values of all indicators.

Initially, disincentives are reduced to a comparable form of disincentives as a unit divided by the disincentive. After that, the input data are standardized (normalized) using the Statistica 10 and Statistica Portable software tools using the “Data”—“Standardize” function. 

Furthermore, to define the integral indicator of the level of healthcare system development in European countries, we carry out a convolution procedure that allows us tp calculate the integral indicators for each group (social, demographic, economic, behavioral) in the context of each year as well as the general integral indicator for each year (Formula (4)) (the analysis period was from 2000 to 2020 for Austria, Belgium, Germany, Denmark, Finland, France, Great Britain, Norway, and Sweden):

(4) $$R_{T}=\sqrt[{\sum f_{i}}]{\overset{m}{\underset{i=1}{\prod }}S_{i}^{f_{i}}},$$

where $R_{T}$ is an integral indicator of the level of healthcare system development of European countries;

$\sum f_{i}$ is the sum of the frequencies; 

$f_{i}$ is the frequency of the studied value (variant) for the i-indicator;

$S_{i}$ is the studied indicator ($i=\bar{1,m}$).

From an economic point of view, the interpretation of the calculations is as follows: the higher the value of the calculated integral indicators, the better the level of healthcare system development. The overall integral indicators take values from 0 to 1. 

In the factor analysis stage, the integral indicators for each group (social, demographic, economic, behavioral) in the context of each year and the general integral indicator of the level of development of the European countries’ healthcare systems each year are output. The performance indicators include the arithmetic means by year in the context of each country and by country in the context of each year.

## 3. Results

In the first stage, the statistical base of the study was formed and modeled in the form of four groups of indicators, behavioral, social, demographic, and economic, using descriptive analysis. A group of 10 European countries was identified (Austria, Belgium, Germany, Denmark, Finland, France, the United Kingdom, the Netherlands, Norway, and Sweden) using a cluster analysis based on the application of an iterative divisive k-means method. 

Multiple units of indicators are determined by the factor analysis of indicators separately for each group (list and units of measurement of indicators of social, demographic, economic, and behavioral features; results are indicated in the article Section on the first stage) in the context of each of the 10 European countries (Austria, Belgium, Germany, Denmark, Finland, France, Great Britain, the Netherlands, Norway, Sweden) for each year of study (from 2000 to 2020).

Evaluation indicators were selected for the period from 2000 to 2020. Thus, in the proposed modeling package, the following statistical data were selected to implement quantitative formalization and as indicators to be input intothe models:

1. Behavioral indicators (indicators that mainly characterize the result of healthcare system functioning, as well as internal factors formed mainly under the influence of personal factors, spirituality, worldview): Incentives: B0—Life expectancy at birth, total (years); B1—Hospital beds (per 1000 people); B2—Immunization, DPT (% of children ages 12–23 months); B3—Immunization, measles (% of children ages 12–23 months). Disincentives: B4—Risk of catastrophic expenditure for surgical care (% of people at risk).
2. Social indicators (indicators that usually characterize the result of healthcare system functioning, environmental factors, socio-cultural indicators formed mainly under the influence of environmental factors and human lifestyle): Incentives: S0—Population growth (annual%); S1—Birth rate, crude (per 1000 people); S10—Fertility rate, total (births per woman); S11—Population, total. Disincentives: S2—Death rate, crude (per 1000 people); S3—Incidence of tuberculosis (per 100,000 people); S4—Maternal mortality ratio (modeled estimate, per 100,000 live births); S5—Mortality caused by road traffic injury (per 100,000 population); S6—Mortality rate, infant (per 1000 live births); S7—Mortality rate, neonatal (per 1000 live births); S8—Mortality rate, under 5 (per 1000 live births); S9—Prevalence of anemia among children (% of children ages 6–59 months).
3. Demographic data (indicators that generally characterize the result of healthcare system functioning, formed depending on gender, age, and population movement): Incentives: D0—Age dependence ratio (% of working-age population); D2—Life expectancy at birth, female (years), D3—Life expectancy at birth, male (years); D4—Population aged 15–64 (% of the total population); D5—Population aged 65 and above (% of the total population); D6—Population, female (% of the total population); D7—Refugee population by country or territory of asylum; D8—Refugee population by country or territory of origin; Disincentive: D1—Adolescent fertility rate (births per 1000 women ages 15–19);
4. Economic indicators (usually acting as factors influencing the healthcare system functioning, formed as a reflection of economic statistics, the evolution of the financial situation, and the revenue and expenditure): Incentives: E0—GDP per capita (current USD); E1—Government expenditure on education, total (% of GDP); E2—Government expenditure per student, territory (% of GDP per capita); E4—Research and development expenditure (% of GDP); E5—GNI per capita, Atlas method (current USD). Disincentive: E3—Poverty gap at USD 1.90 a day (2011 PPP) (%).

Ten European countries were selected for the study: Austria, Belgium, Germany, Denmark, Finland, France, the United Kingdom, the Netherlands, Norway, and Sweden. Four key indicators were initially selected as a statistical basis for further clustering of countries: behavioral—B0 (Life expectancy at birth, total (years)); social—S0 (Population growth (annual %)); demographic—D0 (Age dependency ratio (% of working-age population)); and economic—E0 (GDP per capita (current USD)). An aalysis of the results of the clustering of European countries into three, four, and five clusters determined the adequacy of the three-cluster grouping of countries (Table 2).

Thus, three separate clusters were identified (Table 3 and Table 4) containing a grouping of European countries according to the selected key indicators in a systematic graphical form, indicating the number of member countries of each cluster. Euclidean distances from the grouping center as the defining metric of this type of grouping of European countries. 

Analyzing the clusters of European countries shows that the grouping fully corresponds to the overall level of development of the public health system in countries from the same cluster. Thus, the smallest cluster includes three countries, the average-sized cluster includes 10 countries, and the largest includes 29 countries. According to the number, composition, socio-economic development, and best practices of the countries in the groups, the second cluster was selected to define the countries for the study: Austria, Belgium, Germany, Denmark, Finland, France, the United Kingdom, the Netherlands, Norway, and Sweden.

In the second stage, the degree and significance of the interrelations between the components characterizing the studied groups of indicators (behavioral, social, demographic, economic) were determined using canonical correlations that form an indicator of the level of healthcare system development in European countries. 

Panel data were generated as a table of input data for the period from 2000 to 2020 for 10 European countries selected according to cluster analysis (Austria, Belgium, Germany, Denmark, Finland, France, the United Kingdom, the Netherlands, Norway, and Sweden). The dependence between groups of indicators was alternately determined in the form of six tables (Table 5, Table 6, Table 7, Table 8 and Table 9): behavioral–social, behavioral–demographic, social-economic, demographic-economic, social–demographic, and behavioral–economic.

The data analysis shown in Table 5 determined, first of all, that the analysis is adequate since the value of the canonical correlation coefficient is R = 0.96512, which is close to 1, the value of the adequacy criteria ${\mathrm{Chi}}^{2}$ = 1180 is large enough to aim for infinity, and the value of the probability of the deviation of the hypothesis *p* = 0.0000 tends to 0. Similarly, the adequacy of the analysis is confirmed in other cases in this study (Table 6, Table 7, Table 8 and Table 9). Second, the total redundancy for groups of behavioral indicators is 60.1847% and is 43.9606%for groups of social indicators, which indicates the existence of a relationship between behavioral and social groups of indicators, that is, an average influence of the group of social indicators on the group of behavioral indicators (behavioral indicators depend on social indicators, where social indicators are the cause and behavioral indicators are the consequence).

The analysis of indicators in Table 6 also indicates that there are average-strength relationships between groups of behavioral–demographic indicators (the total redundancy for the group of behavioral indicators is 59.8568% and is 53.0232% for the group of demographic indicators, i.e., behavioral indicators depend on demographic indicators, so demographic indicators are the cause and behavioral indicators are the consequence) and social-economic indicators (the total redundancy for the group of social indicators is 42.0778% and is 59.8402% for the group of economic indicators, i.e., there is an average impact of the group of social indicators on the group of economic indicators, where social indicators are the cause and economic indicators are the effect).

The analysis of indicators in Table 7 indicates that there is a weak relationship between the groups of demographic and economic indicators. In Table 7, the total redundancy for the demographic indicator group is 31.4496% and is 48.3923% for the economic indicator group, i.e., economic indicators depend on demographic indicators, where demographic indicators are the cause and economic indicators are the consequence.

Special attention should be paid to the analysis of indicators in Table 8, which indicates that there is a strong relationship between groups of social and demographic indicators. In Table 8, the total redundancy for the group of social indicators is 69.9279% and is 72.7378% for the group of demographic indicators, i.e., demographic indicators depend on social indicators, where social indicators are the cause and demographic indicators are the consequence.

According to the indicators of Table 9 (where the total redundancy for the group of behavioral indicators is 30.7998% and is 27.7405% for the group of economic factors indicators), it is concluded that the relationship between the groups of behavioral and economic indicators is either a two-way relationship, where behavioral indicators affect the economic ones and the economic indicators affect behavioral ones, or the data should be taken with a time lag.

Thus, we emphasize that special attention in further research should be paid to the groups of social and demographic indicators, which, according to canonical analysis, are indicators and causes that significantly impact the corresponding groups of indicators.

The third stage of factor modeling, conducted by applying the analysis of the principal components, made it possible to determine the relevant indicators for assessing the level of development of the healthcare system in European countries, allowing us to build which were the integral indicators.

**Figure 1:**
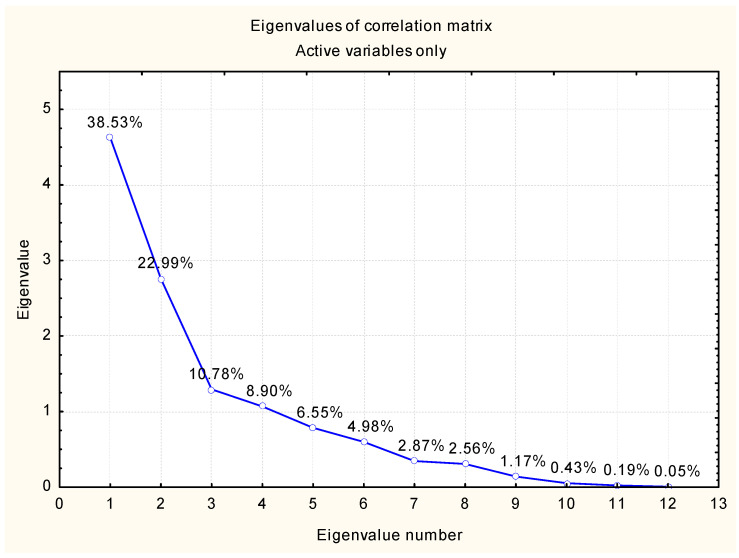
Scree plot of eigenvalues of the correlation matrix of the input data of the group of social indicators for assessing the level of healthcare system development in European countries. Source: independently developed by the authors with Statistica 10 and Statistica Portable.

Thus, a scree plot of the eigenvalues of the correlation matrix of the input data was built to assess the social data of the level of healthcare system development in Europe (Figure 1) in the context of Austria, Belgium, Germany, Denmark, Finland, France, the United Kingdom, the Netherlands, Norway, and Sweden. We formed a table of eigenvalues of the correlation matrix and derived statistical indicators of the social group to assess the level of healthcare system development in European countries (Table 10).

**Figure 2:**
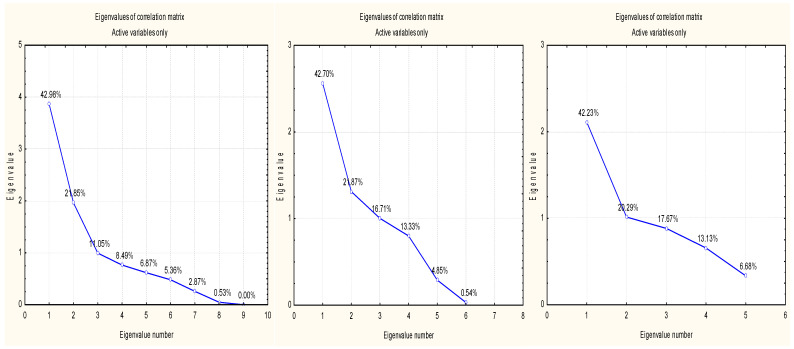
Scree plot of eigenvalues of the correlation matrix of the input data of the group of demographic, economic, and behavioral indicators for assessing the level of healthcare system development in European countries. Source: independently developed by the authors with Statistica 10 and Statistica Portable.

Similarly, scree plots of the eigenvalues of the correlation matrix of the input data of the groups of demographic, economic, and behavioral indicators have been built to assess the level of the healthcare system development in European countries (Figure 2); the “Multivariate Exploratory Techniques”, “Principal Components and Classification Analysis”, and “Eigenvalues” functions were used to form a table of eigenvalues of the correlation matrix and derive statistical demographic, economic, and behavioral indicators to assess the level of healthcare system development in European countries (Table 11).

An analysis of the eigenvalues of the correlation matrix of the input data in the indicators of the social group for assessing the level of the healthcare system development in European countries provides an opportunity to confirm that the first three factors should be taken to define the relevant indicators (together, they account for at least 70% of the variation of the resulting characteristic). These factors are as follows: factor 1—38.52%; factor 2—22.99%; and factor 3 —10.77%. Then, we form a table of variable indicators of the social group to assess the level of healthcare system development in European countries (Table 12).

Like the indicators of the social group, the relevant indicators for each group of indicators (demographic, economic, behavioral) were determined, and the contribution of variables for evaluating the level of healthcare system development in European countries was determined for each group of indicators (Table 13).

Next, intermediate values were obtained to calculate the relevance of indicators in the social group in assessing the level of healthcare system development in European countries (Table 14).

Similarly to the social group, intermediate values for calculating relevance for each group of indicators (demographic, economic, behavioral) were obtained (Table 15, Table 16 and Table 17).

Thus, based on the data in Table 14, Table 15, Table 16 and Table 17, we can conclude that among the indicators of the social group, the most important indicators with the greatest impact on the formation of an effective indicator of the level of healthcare system development in European countries are as follows: S6—Mortality rate, infant (per 1000 live births) and S8—Mortality rate, under 5 (per 1000 live births), accounting for11% of the total impact in the group; S1—Birth rate, crude (per 1000 people) and S7—Mortality rate, neonatal (per 1000 live births), accounting for 10% each. Indicators with an average impact are: S4—Maternal mortality ratio (modeled estimate, per 100,000 live births), S9—Prevalence of anemia among children (% of children ages 6–59 months), and S10—Fertility rate, total (births per woman), accounting for 9% of the total impact in the group each; S3—Incidence of tuberculosis (per 100,000 people) and S5—Mortality caused by road traffic injury (per 100,000 population), accounting for 8% each; and S0—Population growth (annual %), accounting for 7%. The least important indicators are S2—Death rate, crude (per 1000 people), accounting for 5%, and S11—Population, total, accounting for 3%. 

The demographic indicators with the greatest influence on the formation of an effective indicator are as follows: D0—Age dependency ratio (% of working—age population) and D4—Population ages 15–64 (% of the total population), accounting for 13% of total influence in the group; D3—Life expectancy at birth, male (years), D6—Population, female (% of the total population), and D7—Refugee population by country or territory of asylum, accounting for 12% each. Indicators with an average impact are: D5—Population ages 65 and above (% of the total population) and D8—Refugee population by country or territory of origin, accounting for 11% of the total impact in the group; D2—Life expectancy at birth, female (years), accounting for 10%. The least important indicator is D1—Adolescent fertility rate (births per 1000 women ages 15–19), accounting for 7%.

The economic indicators with the greatest impact on the formation of an effective indicator are as follows: E5—GNI per capita, Atlas method (current USD), accounting for 20% of the total impact in the group; E0—GDP per capita (current USD) and E3—Poverty gap at USD 1.90 a day (2011 PPP) (%), accounting for 19% each; and E1—Government expenditure on education, total (% of GDP), accounting for 17%. E2—Government expenditure per student, territory (% of GDP per capita) had an average impact, accounting for 14% of the total impact in the group. The least important indicator is E4—Research and development expenditure (% of GDP), accounting for 11%.

The behavioural indicators with the greatest impact on the formation of an effective indicator are: B1—Hospital beds (per 1000 people), accounting for 21% of the total impact in the group; B2—Immunization, DPT (% of children ages 12–23 months), B3—Immunization, measles (% of children ages 12–23 months), and B0—Life expectancy at birth, total (years), accounting for 20% each. E1–B4—Risk of catastrophic expenditure for surgical care (% of people at risk) had an average impact; indicators with no impact were not detected among the behavioral group.

We propose that the most influential indicators should be considered in further studies.

Disincentives were reduced to a comparable form. After, the input data were standardized (normalized) (Table 18). 

Each country’s integral indicators are calculated separately (social, demographic, economic, behavioral) each year (Table 19, Table 20, Table 21 and Table 22), as well as arithmetic mean by year in the context of countries and by country in the context of years. 

The economic interpretation of the calculations performed for the group of integral social indicators (i.e., those that act both as a result and as factors influencing the functioning of the health system) shows that the following countries have the highest average values of integral social indicators according to Table 19: Norway 0.75, the United Kingdom 0.73, Finland 0.67. These values indicate the best states of social indicators that determine the level of health system development in countries. (High values of these indicators and their positive dynamics in recent years confirm this: Norway for 2020—1.68; Finland for 2020—1.47. However, this indicator for the United Kingdom for 2020 was 0.61, requiring additional attention due to its unstable dynamics.) Average values were found for Germany 0.59, France 0.54, and Sweden 0.54. (High values of these indicators and their relatively positive dynamics in recent years confirm this: Germany for 2020—0.70; Sweden for 2020—0.63. However, France for 2020 scored 0.36 for this indicator, which is low and indicates unstable dynamics with existing problems). The lowest, but not critical, values are observed for the Netherlands—0.46; Belgium—0.45; Austria—0.44; and Denmark—0.4, indicating shortcomings in the state of social indicators for these countries, which requires additional attention. 

For the group of integral demographic indicators that act both as results and as factors influencing the functioning of the health system, the economic interpretation of calculations shows that, according to Table 20, the largest average values are observed for Germany 0.64, Finland 0.62, and Denmark 0.61; these values indicate the best state of demographic indicators that determine the level of health system development in the studied countries. (High values of these indicators in recent years and their positive dynamics confirm this: Denmark for 2020—0.84; Finland for 2020—0.82. However, this indicator for Germany for 2020 was 0.47, which requires additional attention due to its unstable dynamics.) Average values were found for the countries France (0.59), Austria (0.58), and Sweden (0.54), and below average values were found for the Netherlands (0.49), Norway (0.49), and the United Kingdom (0.44). It should be noted that, among these countries, the countries with the highest levels of the integral indicators in recent years were France (1.13) and Sweden (1.08), which indicates a particularly positive trend in these countries. The average values of integral demographic indicators are confirmed by the average values of these indicators in recent years and their relatively positive dynamics: the Netherlands for 2020—0.50; Norway for 2020—0.41. However, this indicator for Austria for 2020 was 0.22, and 0.21 for the United Kingdom thus indicating unstable dynamics and existing problematic aspects. The lowest value was found in Belgium at 0.36, which shows shortcomings in the state of demographic indicators, requiring additional attention. 

For a group of integral economic indicators that act both as factors of influence and as a result of the functioning of the health system, the economic interpretation of the calculations shows that, according to Table 21, the largest average value of the integral economic indicator is observed for Norway at 1.01. High values are observed in Denmark (0.84), Sweden (0.71), the United Kingdom (0.68), and the Netherlands (0.62); these values indicate the best state of economic indicators that determine the level of health system development in the studied countries. (This is confirmed by the high values of these indicators and their relatively positive dynamics in recent years: Norway for 2020–0.79; Denmark for 2020–0.71; Sweden for 2020–0.77; the United Kingdom for 2020–0.67; the Netherlands for 2020–0.54. However, Denmark, Norway, and Sweden have unstable dynamics, which requires additional attention.) An average value is observed for France (0.58), and below average for Austria (0.45), Finland (0.44), and Germany (0.43) (the values of these indicators for recent years: France for 2020–0.53; Austria for 2020–0.41; Finland for 2020–0.41; and Germany had a low indicator for 2020—0.36—and unstable dynamics, which indicates existing problematic aspects). The lowest value (Belgium—0.37) indicates shortcomings in the state of economic indicators, which requires additional attention. 

The economic interpretation of the calculations performed for the group of integral behavioral indicators, which act both as results and as factors influencing the functioning of the health system, shows that, according to Table 22, the highest average value of the integral behavioral indicator was found in Austria (0.96) High values were observed in France (0.78), Sweden (0.73), Finland (0.68), Denmark (0.65), and the United Kingdom (0.61), which indicates the best state of behavioral indicators that determine the level of healthcare system development in the studied countries. (High values of these indicators and their positive dynamics in recent years confirm this: Austria for 2020—0.72; France for 2020—0.62; Sweden for 2020—0.84; Finland for 2020—0.77; Denmark for 2020—0.57. However, for the United Kingdom for 2020, a value of 0.45 was obtained, requiring additional attention.) Average values are observed for Germany at 0.58, Belgium at 0.58 (the average values of these indicators in recent years and their relatively positive dynamics confirm this: Germany for 2020—0.57; Belgium for 2020—0.49). The lowest, but not critical, values are observed for the Netherlands at 0.48 and Norway at 0.46, which indicate shortcomings in the state of social indicators, requiring additional attention. However, we note the positive dynamics for Norway, the highest indicator for 2020 being 0.79.

The general integral indicators are derived together for all countries’ indicators each year, as well as by the arithmetic mean for years in the context of countries and for countries in the context of years.

Thus, the economic interpretation of the calculations performed for complex integral indicators shows that, according to Table 23, the highest average values of complex integral indicators are observed for Norway at 0.61 and Sweden at 0.60, indicating the best state of indicators that define the highest level of healthcare system development in these countries among the selected cluster of countries (high values of these indicators in recent years and their positive dynamics for the period from 2000 to 2020 confirm this: Norway for 2020—0.81; Sweden for 2020—0.8). Average levels of integral indicators were found in the following countries: France at 0.59, the United Kingdom at 0.58, Denmark at 0.58, Finland at 0.56, Austria at 0.56, Germany at 0.54, and the Netherlands at 0.50. This indicates the good state of indicators that show the level of health system development in these countries. (The high values of these indicators in recent years and their positive dynamics for the period from 2000 to 2020 also confirm this: France for 2020—0.60; Denmark for 2020—0.62; Finland for 2020—0.79; Germany for 2020—0.51; and the Netherlands for 2020—0.51. However, the United Kingdom for 2020 was at 0.44 and Austria for 2020 was at 0.41, indicating their unstable dynamics and insufficiently high level of indicators in comparison to other countries of the cluster; they need additional attention.) Belgium has the lowest but not a critical value of 0.41; this indicates shortcomings in the state of indicators that determine the level of health system development in the country and requires additional attention. 

A high level of integral indicators indicates the positive development of the health care system in the country, and a low level of integral indicators for the state is an alarming indicator since this shows a low level of healthcare system development. Therefore, the representatives of healthcare management should regularly, carefully, and continuously monitor the factors that lead to a decrease in integral indicators and take appropriate measures.

## 4. Discussion

This study refers to proposals to improve healthcare system by further developing the approach to qualitative and quantitative assessments of health system indicators by developing an integral indicator in the context of behavioral, social, demographic, and economic factors that characterize the level of the health system development in European countries using the selected methods of multidimensional statistical modeling.

The steps of this complex scientific study of behavioral and social analysis of the public health system were based on the the work of the authors [90] in the health care field. As a result of this study of the behavioral and social dimensions of the public health systems of the world based on the use of bibliometric analysis, the main scientific categories of the study were determined, the most potential priority areas of policy and strategy formation of the healthcare system were identified, and the territorial component was theoretically determined for further considerations.

In addition, the previous works by the authors of [46] used the method of clustering countries into specific groups, which confirmed the appropriateness of the obtained distribution. This technique was applied to group European countries into appropriate clusters for further analysis in the current study. Previous healthcare research by the authors of [47] studied the factors of the healthcare system and their influence on the vulnerability of the population of a certain region; relevant factors are identified.

The analysis of literary sources shows the practicality of using the selected types of analysis—descriptive analysis, cluster analysis, canonical analysis, and factor analysis—for research in the healthcare field. Moreover, descriptive analysis allows various relevant indicators available in databases to be combined into appropriate groups. With the help of cluster analysis, it is possible to adequately divide countries into corresponding comparable research groups. Canonical analysis makes it possible to determine the relationships between the component characteristics of the studied groups of indicators and the nature of such relationships. By conducting a factor analysis, the relevant indicators of the studied indicator are determined, and the integrated indicator of the selected indicators is modeled.

The authors’ contributions to the knowledge of healthcare system include:

The determination of the degree and significance of interrelations between groups of behavioral, social, demographic, and economic indicators of the level of healthcare system development in European countries based on the use of canonical analysis in terms of using canonical correlations;

The definition of relevant indicators to assess the level of healthcare system development in Europe based on the use of factor analysis in the form of principal component analysis;

The construction of integral indicators of the level of healthcare system development in Europe in the context of behavioral, social, demographic, and economic factors based on factor modeling and using the convolution procedure.

The authors’ approaches to the behavioral and social measurement of the public health system were developed separately. These approaches so not act as one full-fledged integrated approach to assess the level of health system development since the study uses a limited list of indicators selected by the author in the context of behavioral, social, demographic, and economic factors characterizing the state of healthcare systems. However, the developed approach is the best addition to the existing systems for measuring the state of health systems in European countries because this approach: 

Conducts a statistical assessment of the significance and relationship of the studied characteristics; that is, it conducts a causal analysis of groups of behavioral, social, demographic, and economic indicators of the level of health system development in European countries, based on canonical analysis, which, in contrast to existing approaches, takes into account canonical correlations between groups of indicators;

Determines the significance of factors within each group of behavioral, social, demographic, and economic indicators for assessing the level of the European health system development based on factor analysis; the method of analyzing principal components was used to effectively determine relevant indicators in other scientific fields;

Identifies integral indicators of the level of health systems development in European countries by groups of behavioral, social, demographic, and economic factors, based on factor modeling, which allows the use of procedures and convolution to quickly determine common general indicators for each group and a common integral indicator, greatly facilitating further analysis.

The validity of the results is confirmed by the fact that the chosen research methodologies, namely, descriptive, canonical, and factor analyses, are reasonably suitable for study of the state of European health systems and achieved the set goal of the study. The calculated data obtained correspond to the actual situation and dynamics of the healthcare sector for 2000–2020.

## 5. Conclusions and Recommendations

The descriptive analysis made it possible, first, to form and model a multi-aspect array of groups of behavioral, social, demographic, and economic indicators, which are relevant for the further formation of an integral indicator of the level of healthcare system development in European countries. Second, based on cluster analysis, which involves the application of the iterative divisive k-means method, it was possible to determine a group of 10 European countries for the study (Austria, Belgium, Germany, Denmark, Finland, France, the United Kingdom, the Netherlands, Norway, and Sweden). 

The canonical analysis specifies the degree and significance of relations between the constituent features of the studied groups of indicators to establish systems of pairs of features that are most correlated with each other and form an indicator of the level of healthcare system development in European countries. Such indicators and reasons that significantly impact the corresponding groups of indicators are the components of the groups of social and demographic indicators.

Considering the results of the canonical analysis research stage, researchers should take into account the most correlated pairs of features to form narrowly concentrated combinations of relationships. At the same time, practitioners should focus on casual and effective characteristics. Thus, it is recommended to consider social characteristics that affect behavioral, economic, and demographic indicators; demographic characteristics that affect economic indicators; and two-way communication between groups of behavioral and economic indicators.

The factor analysis, conducted by applying the study of the principal components, made it possible to determine the relevant indicators for evaluating the level of healthcare system development in European countries (i.e., to establish the importance of factors in the middle of each group of indicators). The most relevant indicators among the indicators of the social group were Mortality rate (infant), Mortality rate (under 5), Birth rate (crude), and Mortality rate (neonatal). The most relevant indicators among the demographic group were Age dependency ratio, Population ages 15–64, Life expectancy at birth (male), Population (female), and Refugee population by country or territory of asylum. Among the economic indicators, the most important were GNI per capita, GDP per capita, and poverty gap at USD 1.90 a day. Finally, the most important indicators of the behavioral group were Hospital beds, Immunization (DPT), Immunization (measles), and Life expectancy at birth (total).

Factor analysis allowed for building an integral indicator of the level of healthcare system development of European countries. The highest average values of integral social indicators are observed for the following countries: Norway (0.75), the United Kingdom (0.73), and Finland (0.67); the highest integral demographic indicators were found in Germany (0.64), Finland (0.62), and Denmark (0.61); the highest integral economic indicator were found in Norway (1.01), Denmark (0.84), Sweden (0.71), the United Kingdom (0.68), and the Netherlands (0.62); the highest integral behavioral indicators were found in Austria (0.96), France (0.78), Sweden (0.73), Finland (0.68), Denmark (0.65), and the United Kingdom (0.61); and the highest comprehensive integral indicators were found in Norway (0.61) and Sweden (0.60). These values indicate the best state of indicators that determine the level of the health system development in the countries. The lowest values of integral social indicators are observed for the following countries: The Netherlands (0.46), Belgium (0.45), Austria (0.44), Denmark (0.41); integral demographic indicator–Belgium (0.36); integral economic indicator–Belgium (0.37); integral behavioral indicator–The Netherlands (0.48), Norway (0.46); complex integral indicator in Belgium (0.41)-indicates shortcomings in the state of indicators, which requires additional attention. 

Considering the results of the factor analysis, it is recommended that future studies focus on a deeper study of certain relevant indicators, which have the greatest impact on the group. Therefore, it is recommended that further studies also analyze them separately to determine the factors that affect the formation of these relevant factors to achieve the maximum level of healthcare system development.

Furthermore, according to the results of factor analysis, it is recommended to analyze in detail the levels of integral indicators in dynamics in the context of each country. It is first recommended to study the integral indicators that comprehensively cover the overall level of healthcare system development and provide a comprehensive picture of the direction under study. At the same time, specialists are recommended to focus on integral indicators, the levels of which, as indicated in the study, have the lowest values and unstable or negative dynamics.

Thus, the results were summarized using the following indicators: the integral indicators for each group (social, demographic, economic, behavior) in the context of each year and the general integral indicator of the level of healthcare system development of European countries for each year; the arithmetic mean by year in the context of each country and by country in the context of years. Other generalizations are not provided by the proposed model.

When constructing the model, we considered explicit hypotheses of the presence of relationships between the indicators of the behavioral and social, behavioral and demographic, behavioral and economic, social and demographic, and economic, demographic, and economic groups. Non-explicit hypotheses were ignored.

The need to improve the level of healthcare system development in European countries was confirmed. Shortcomings and possible reserves for potential improvement of the healthcare system were identified based on the statistically significant models and by analyzing relevant factors.

Therefore, for the effective development of the healthcare system in the countries of Europe and of the world, it is advisable to review theoretical approaches and practical results of the state of development of the medical system on an ongoing basis, taking into account the dynamic changes in the influencing factors that determine the priorities of this field.

The results of this study will help managers and organizers of the public health system of European countries to use the received factual and analyzed data in making managerial decisions; to build strong, substantiated links between the available objective data and the policies of the healthcare system, the level of the healthcare system development in European countries, and positive changes in the health sector.

The study’s results can help government officials, employees of the healthcare sector, and those providing medical services identify possible reserves for potential improvement of the level of the health system based on developed statistically significant models, as well as by analyzing relevant factors. 

Based on the research results, state authorities can conduct effective, timely, high-quality regulation and adjustment of the regulatory and legislative framework to improve the level of healthcare system development in European countries and the healthcare system.

## Figures and Tables

**Table 1:** The definition of the source database for canonical analysis.

Observation Sequence Number	System of Attributes of the First Group				System of Attributes of the Second Group			
	$A_{1}$	$A_{2}$	…	$A_{x}$	$B_{1}$	$B_{2}$	…	$B_{y}$
1	$a_{11}$	$a_{12}$	…	$a_{1x}$	$b_{11}$	$b_{12}$	…	$b_{1y}$
…	…	…	…	…	…	…	…	…
N	$a_{n1}$	$a_{n2}$	…	$a_{\mathrm{nx}}$	$b_{n1}$	$b_{n2}$	…	$b_{\mathrm{ny}}$

Source: independently developed by the authors with Statistica 10 and Statistica Portable.

**Table 2:** Analysis of the adequacy of clustering European countries into three groups as of 2020.

Variable	Analysis of Variance (Spreadsheet1.sta)					
	Between SS	df	Within SS	df	F	signif. p
B0	$2.206909\;\times \;10^{2}$	2	$3.211732\;\times \;10^{2}$	39	13.3992	0.000037
S0	$4.013948\;\times \;10^{0}$	2	$1.654163\;\times \;10^{1}$	39	4.7318	0.014459
D0	$2.315282\;\times \;10^{2}$	2	$9.411579\;\times \;10^{2}$	39	4.7971	0.013721
E0	$2.408162\;\times \;10^{10}$	2	$3.460462\;\times \;10^{9}$	39	135.7020	0.000000

Source: independently developed by the authors with Statistica 10 and Statistica Portable.

**Table 3:** Composition and characteristics of the first and second of the three conditional clusters of European countries in the context of the public health system according to the Euclidean distance indicator.

Members of Cluster Number 1 (Spreadsheet and Distances from Respective Cluster). Cluster Contains 3 Cases		Members of Cluster Number 2 (Spreadsheet and Distances from Respective Cluster). Cluster Contains 10 Cases	
	Distance		Distance
Switzerland	4596.31	Austria	826.476
Ireland	5435.25	Belgium	2526.116
Luxemburg	10031.56	Germany	1994.455
		Denmark	5410.859
		Finland	540.387
		France	5602.239
		The United Kingdom	4571.760
		The Netherlands	1077.217
		Norway	8544.010
		Sweden	1029.306

Source: independently developed by the authors with Statistica 10 and Statistica Portable.

**Table 4:** Composition and characteristics of the third of the three conditional clusters of European countries in the context of the public health system according to the Euclidean distance indicator.

Members of Cluster Number 3 (Spreadsheet and Distances from Respective Cluster). Cluster Contains 29 Cases			
	Distance		Distance
Albania	4651.189	Lithuania	2756.454
Armenia	5184.260	Latvia	1534.712
Azerbaijan	5202.316	Moldova	5054.394
Bulgaria	2277.669	North Macedonia	4394.038
Bosnia and Herzegovina	4276.086	Malta	7155.963
Cyprus	6523.516	Montenegro	3469.952
Czech Republic	4149.481	Poland	553.958
Spain	6210.942	Portugal	3780.015
Estonia	4209.912	Romania	838.987
Georgia	5189.398	Serbia	3451.923
Greece	1506.350	Slovak Republic	2315.989
Croatia	251.033	Slovenia	5427.482
Hungary	720.718	Turkiye	3049.053
Italy	8600.218	Ukraine	5441.399
Kazakhstan	2756.454		

Source: independently developed by the authors with Statistica 10 and Statistica Portable.

**Table 5:** Relations between behavioral and social groups of indicators between behavioral and demographic groups of indicators.

N = 210	Canonical Analysis Summary (Spreadsheet) Canonical R: 0.96512 ${\mathrm{Chi}}^{2}(60)=1180.3\;p=0.0000$	
	Left Set	Right Set
No. of variables	5	12
Variance extracted	100.000%	71.7945%
Total redundancy	60.1847%	43.9606%
Variables: 1	B1	S1
2	B2	S2
3	B3	S3
4	B0	S4
5	B4	S5
6		S6
7		S7
8		S8
9		S0
10		S9
11		S10
12		S11

Source: independently developed by the authors with Statistica 10 and Statistica Portable.

**Table 6:** Relations between behavioral and demographic groups of indicators between social and economic groups of indicators.

N = 210	Canonical Analysis Summary (Spreadsheet) Canonical R: 1.0000 ${\mathrm{Chi}}^{2}(45)=0.0000\;p=0.0000$		N = 210	Canonical Analysis Summary (Spreadsheet) Canonical R: 0.92055 ${\mathrm{Chi}}^{2}(72)=920.02\;p=0.0000$	
	Left Set	Right Set		Left Set	Right Set
No. of variables	5	12	No. of variables	12	6
Variance extracted	100.000%	75.9071%	Variance extracted	66.8391%	100.000%
Total redundancy	59.8568%	53.0232%	Total redundancy	42.0778%	59.8402%
Variables: 1	B1	D1	Variables: 1	S1	E0
2	B2	D0	2	S2	E1
3	B3	D2	3	S3	E2
4	B0	D3	4	S4	E3
5	B4	D4	5	S5	E4
6		D5	6	S6	E5
7		D6	7	S7	
8		D7	8	S8	
9		D8	9	S0	
			10	S9	
			11	S10	
			12	S11	

Source: independently developed by the authors with Statistica 10 and Statistica Portable.

**Table 7:** Relations between demographic and economic groups of indicators.

N = 210	Canonical Analysis Summary (Spreadsheet) Canonical R: 0.83026 ${\mathrm{Chi}}^{2}(54)=687.32\;p=0.0000$	
	Left Set	Right Set
No. of variables	9	6
Variance extracted	79.5333%	100.000%
Total redundancy	31.4496%	48.3923%
Variables: 1	D1	E0
2	D0	E1
3	D2	E2
4	D3	E3
5	D4	E4
6	D5	E5
7	D6	
8	D7	
9	D8	

Source: independently developed by the authors with Statistica 10 and Statistica Portable.

**Table 8:** Relations between social and demographic indicators.

N = 210	Canonical Analysis Summary (Spreadsheet) Canonical R: 0.97477 $Chi^{2}$(108) = 2268.5 *p* = 0.0000	
	Left Set	Right Set
No. of variables	12	9
Variance extracted	91.3868%	100.000%
Total redundancy	68.9279%	72.7278%
Variables: 1	S1	D1
2	S2	D0
3	S3	D2
4	S4	D3
5	S5	D4
6	S6	D5
7	S7	D6
8	S8	D7
9	S0	D8
10	S9	
11	S10	
12	S11	

Source: independently developed by the authors with Statistica 10 and Statistica Portable.

**Table 9:** Relations between behavioral and economic groups of indicators.

N = 210	Canonical Analysis Summary (Spreadsheet) Canonical R: 0.66013 $Chi^{2}$(30) = 356.20 *p* = 0.0000	
	Left Set	Right Set
No. of variables	9	6
Variance extracted	100.000%	88.6172%
Total redundancy	30.7998%	27.7405%
Variables: 1	B1	E0
2	B2	E1
3	B3	E2
4	B0	E3
5	B4	E4
6		E5

Source: independently developed by the authors with Statistica 10 and Statistica Portable.

**Table 10:** Eigenvalues of the correlation matrix and derived statistical indicators of the social group for assessing the level of the healthcare system development in European countries.

Value Number	Eigenvalues of Correlation Matrix, and Related Statistics (Spreadsheet1.sta) Active Variables Only			
	Eigenvalue	% Total Variance	Cumulative Eigenvalue	Cumulative%
1	4.623404	38.52836	4.62340	38.528
2	2.759080	22.99233	7.38248	61.520
3	1.293385	10.77821	8.67587	72.298
4	1.067766	8.89805	9.74363	81.197
5	0.785697	6.54748	10.52933	87.744
6	0.597571	4.97976	11.12690	92.724
7	0.344740	2.87284	11.47164	95.597
8	0.307457	2.56214	11.77910	98.159
9	0.139928	1.16607	11.91903	99.325
10	0.051840	0.43200	11.97087	99.757
11	0.022747	0.18956	11.99362	99.946
12	0.006385	0.05321	12.00000	100.000

Source: independently developed by the authors with Statistica 10 and Statistica Portable.

**Table 11:** Eigenvalues of the correlation matrix and derived statistical indicators of the demographic, economic, and behavioral groups for assessing the level of healthcare system development in European countries.

Value Number	Eigenvalues of Correlation Matrix and Related Statistics (Spreadsheet1.sta) Active Variables Only			
	Eigenvalue	% Total Variance	Cumulative Eigenvalue	Cumulative %
1	3.868142	42.97935	3.868142	42.979
2	1.966286	21.84762	5.834427	64.827
3	0.994087	11.04541	6.828514	75.872
4	0.764443	8.49381	7.592957	84.366
5	0.617925	6.86584	8.210883	91.232
6	0.482025	5.35584	8.692908	96.587
7	0.258537	2.87264	8.951445	99.460
8	0.048147	0.53496	8.999592	99.995
9	0.000408	0.00453	9.000000	100.000
Value number	Eigenvalues of correlation matrix and related statistics (Spreadsheet1.sta) Active variables only			
	Eigenvalue	% Total variance	Cumulative Eigenvalue	Cumulative %
1	2.56172	42.6953	2.56172	42.69
2	1.31215	21.8692	3.87387	64.56
3	1.00274	16.7123	4.87661	81.27
4	0.79993	13.3322	5.67655	94.60
5	0.29079	4.8465	5.96734	99.45
6	0.03265	0.5442	6.00000	100.00
Value number	Eigenvalues of correlation matrix and related statistics (Spreadsheet1.sta) Active variables only			
	Eigenvalue	% Total variance	Cumulative Eigenvalue	Cumulative %
1	2.111645	42.23289	2.111645	42.232
2	1.014446	20.28891	3.126090	62.521
3	0.883353	17.66706	4.009443	80.188
4	0.656482	13.12964	4.665925	93.318
5	0.334075	6.68149	5.000000	100.000

Source: independently developed by the authors with Statistica 10 and Statistica Portable.

**Table 12:** The contribution of the variables of the social group for assessing the level of the healthcare system development in Europe.

Variable	Variable Contributions, Based on Correlations (Spreadsheet261.sta)				
	Factor 1	Factor 2	Factor 3	Factor 4	Factor 5
S1	0.018482	0.296107	0.003294	0.008610	0.027638
S2	0.004701	0.152480	0.000405	0.000597	0.558676
S3	0.133997	0.019296	0.003146	0.021732	0.027085
S4	0.156495	0.005455	0.001932	0.078477	0.002037
S5	0.053683	0.011188	0.304215	0.134703	0.089569
S6	0.195382	0.003937	0.007948	0.020499	0.017394
S7	0.174913	0.002146	0.024564	0.011907	0.087386
S8	0.206219	0.001108	0.000385	0.010126	0.010217
S0	0.005657	0.204889	0.037149	0.070963	0.054261
S9	0.004337	0.002111	0.600057	0.056489	0.051134
S10	0.003017	0.276982	0.002799	0.057002	0.049950
S11	0.043117	0.024300	0.014106	0.528897	0.024653

Source: independently developed by the authors with Statistica 10 and Statistica Portable.

**Table 13:** The contribution of variable indicators of the social group to assess the level of the healthcare system development in European countries by a group of indicators (demographic, economic, behavioral).

Variable	Variable Contributions, Based on Correlations (Spreadsheet261.sta)				
	Factor 1	Factor 2	Factor 3	Factor 4	Factor 5
D1	0.077111	0.079548	0.047481	0.260780	0.401003
D0	0.195446	0.017942	0.068445	0.135547	0.018158
D2	0.166072	0.004607	0.008746	0.259701	0.176632
D3	0.174418	0.011564	0.108852	0.001949	0.275718
D4	0.195222	0.017249	0.066438	0.141530	0.017761
D5	0.142477	0.102995	0.003793	0.005688	0.034684
D6	0.024041	0.181176	0.394172	0.194719	0.000042
D7	0.000049	0.325465	0.185161	0.000049	0.054364
D8	0.025164	0.259454	0.116911	0.000037	0.021639
Variable	Variable contributions, based on correlations (Spreadsheet261.sta)				
	Factor 1	Factor 2	Factor 3	Factor 4	Factor 5
E0	0.290626	0.144518	0.011220	0.028360	0.057240
E1	0.259914	0.103360	0.006465	0.021987	0.598959
E2	0.133973	0.202333	0,086272	0.270547	0.303554
E3	0.0014424	0.039787	0.883627	0.071289	0.003474
E4	0.014783	0.369459	0.005006	0.579030	0.031388
E5	0.299262	0.140543	0.007410	0.028787	0.005383
Variable	Variable contributions, based on correlations (Spreadsheet261.sta)				
	Factor 1	Factor 2	Factor 3	Factor 4	Factor 5
B1	0.111294	0.255137	0.399166	0.233405	0.000998
B2	0.3081169	0.143765	0.012472	0.042069	0.493578
B3	0.280694	0.003028	0.246940	0.090793	0.378544
B0	0.121629	0.449543	0.082670	0.305381	0.040778
B4	0.178267	0.148527	0.258751	0.328352	0.086103

Source: independently developed by the authors with Statistica 10 and Statistica Portable.

**Table 14:** Intermediate values for calculating the relevance of social group to assess the level of healthcare system development in European countries.

Indicators/Weighting Coefficients	Factor 1	Factor 2	Factor 3	Weighted Impact of Indicators
	38.52836	22.99233	10.77821	
S1—Birth rate, crude (per 1000 people)	0.018482	0.296107	0.003294	10%
S2—Death rate, crude (per 1000 people)	0.004701	0.152480	0.000405	5%
S3—Incidence of tuberculosis (per 100,000 people)	0.133997	0.019296	0.003146	8%
S4—Maternal mortality ratio (modeled estimate, per 100,000 live births)	0.156495	0.005455	0.001932	9%
S5—Mortality caused by road traffic injury (per 100,000 population),	0.053683	0.011188	0.304215	8%
S6—Mortality rate, infant (per 1000 live births)	0.195382	0.003937	0.007948	11%
S7—Mortality rate, neonatal (per 1000 live births)	0.174913	0.002146	0.024564	10%
S8—Mortality rate, under-5 (per 1000 live births)	0.206219	0.001108	0.000385	11%
S0—Population growth (annual %)	0.005657	0.204889	0.037149	7%
S9—Prevalence of anemia among children (% of children ages 6–59 months)	0.004337	0.002111	0.600057	9%
S10—Fertility rate, total (births per woman)	0.003017	0.276982	0.002799	9%
S11—Population, total	0.043117	0.024300	0.014106	3%

Source: independently developed by the authors.

**Table 15:** Intermediate values for calculating the relevance of indicators in the demographic group for assessing the level of healthcare system development in European countries.

Indicators/Weighting Coefficients	Factor 1	Factor 2	Factor 3	Weighted Impact of Indicators
	42.97935	21.84762	11.04541	
D1—Adolescent fertility rate (births per 1000 women ages 15–19)	0.077111	0.079548	0.047481	7%
D0—Age dependency ratio (% of working-age population)	0.195446	0.017942	0.068445	13%
D2—Life expectancy at birth, female (years)	0.166072	0.004607	0.008746	10%
D3—Life expectancy at birth, male (years)	0.174418	0.011564	0.108852	12%
D4—Population ages 15–64 (% of total population)	0.195222	0.017249	0.066438	13%
D5—Population ages 65 and above (% of total population)	0.142477	0.102995	0.003793	11%
D6—Population, female (% of total population)	0.024041	0.181176	0.394172	12%
D7 —Refugee population by country or territory of asylum	0.000049	0.325465	0.185161	12%
D8—Refugee population by country or territory of origin	0.025164	0.259454	0.116911	11%

Source: independently developed by the authors.

**Table 16:** Intermediate values for calculating the relevance of indicators in the economic group to assess the level of healthcare system development in European countries.

Indicators/Weighting Coefficients	Factor 1	Factor 2	Factor 3	Weighted Impact of Indicators
	42.69537	21.86926	16.71236	
E0—GDP per capita (current USD)	0.290626	0.144518	0.011220	19%
E1—Government expenditure on education, total (% of GDP)	0.259914	0.103360	0.006465	17%
E2—Government expenditure per student, tertiary (% of GDP per capita),	0.133973	0.202333	0.086272	14%
E3—Poverty gap at USD 1.90 a day (2011 PPP) (%)	0.001442	0.039787	0.883627	19%
E4—Research and development expenditure (% of GDP)	0.014783	0.369459	0.005006	11%
E5—GNI per capita, Atlas method (current USD)	0.299262	0.140543	0.007410	20%

Source: independently developed by the authors.

**Table 17:** Intermediate values for calculating the relevance of indicators in the behavioral group to assess the level of healthcare system development in European countries.

Indicators/Weighting Coefficients	Factor 1	Factor 2	Factor 3	Weighted Impact of Indicators
	42.23289	20.28891	17.66706	
B1—Hospital beds (per 1000 people)	0.111294	0.255137	0.399166	21%
B2—Immunization, DPT (% of children ages 12–23 months)	0.308116	0.143765	0.012472	20%
B3—Immunization, measles (% of children ages 12–23 months)	0.280694	0.003028	0.246940	20%
B0—Life expectancy at birth, total (years)	0.121629	0.449543	0.082670	20%
B4—Risk of catastrophic expenditure for surgical care (% of people at risk)	0.178267	0.148527	0.258751	19%

Source: independently developed by the authors.

**Table 18:** A fragment of standardized (normalized) data of indicators of the level of healthcare system development in European countries.

S1	S2	S3	S4	S5	S6	…	D5	D6	D7	D8	E0	E1	E2
−1.1660	−1.0640	1.3452	−0.2972	−1.4369	0.2619	…	0.7484	−0.0036	−0.0829	−0.3895	−1.6709	−0.4223	−0.0976
−0.7753	−1.7254	−0.1799	−0.8397	−1.6515	−0.2275	…	0.7738	−0.5356	−0.4140	−0.3012	−1.5779	−0.2687	−0.4971
−1.2441	−2.4615	0.8876	−0.5712	−1.0890	0.1277	…	2.0016	−0.2738	4.5218	0.0783	−1.5383	−1.6816	0.4470
−0.3847	−0.0688	1.3452	−0.8607	−1.0440	0.1277	…	1.2329	−0.8246	−0.5361	−0.5042	−1.0704	1.9103	3.9410
−1.9472	−0.7521	2.8597	0.0646	−1.0872	2.6707	…	2.4392	−0.0434	−0.5930	−0.4756	−1.4951	−0.3097	−0.0534
0.0060	−0.6440	−0.3427	−1.1243	−1.5224	−0.2275	…	1.5303	1.7714	1.1847	−0.1159	−1.6230	−0.5087	−1.0491
−0.5409	−1.1639	0.1295	−1.2523	−0.7370	−0.4315	…	0.4705	−0.2307	−0.1239	−0.0012	−1.2377	−1.8858	−2.1790
−0.9316	−0.4210	2.1635	−1.4513	−0.7167	−0.4315	…	1.1677	−1.0460	−0.2808	−0.0982	−1.3710	−1.3605	−0.9186
−0.8534	2.8165	3.7804	0.0040	−0.6728	3.0357	…	−0.0981	−2.4633	−0.4800	−0.5042	−0.5799	0.3988	0.1195
0.0060	−0.1887	2.8597	0.1086	−0.7398	2.0451	…	1.3160	−1.5767	0.3761	−0.4777	−1.1446	0.6539	1.2365
…	…	…	…	…	…	…	…	…	…	…	…	…	…
−1.1660	−1.0640	1.3452	0.2301	0.1895	0.2619	…	0.7484	−0.0036	−0.0829	−0.3895	0.3196	−0.7993	−0.2209
−0.7753	−1.7254	−0.1799	0.3735	−0.1958	−0.2275	…	0.7738	−0.5356	−0.4140	−0.3012	−0.1113	0.4075	−0.7681
−1.2441	−2.4615	0.8876	−0.5712	0.9173	0.1277	…	2.0016	−0.2738	4.5218	0.0783	−0.0407	−0.8797	−1.1735
−0.3847	−0.0688	1.3452	1.2021	1.0046	0.1277	…	1.2329	−0.8246	−0.5361	−0.5042	0.9425	0.5814	0.3435
−1.9472	−0.7521	2.8597	2.2290	0.8286	2.6707	…	2.4392	−0.0434	−0.5930	−0.5067	0.1524	0.2814	−0.5819
0.0060	−0.6440	−0.3427	−0.7277	0.0967	−0.2275	…	1.5303	1.7714	1.1847	−0.1159	−0.5198	−0.6742	−0.7054
−0.5409	−1.1639	0.1295	−0.4133	1.5177	−0.4315	…	0.4705	−0.2307	−0.1239	−0.0012	−0.3829	−0.6794	0.8380
−0.9316	−0.4210	2.1635	0.6338	0.7032	−0.4315	…	1.1677	−1.0460	−0.2808	−0.0982	0.3671	−0.5510	1.4721
−0.8534	2.8165	3.7804	5.0655	3.6709	3.0357	…	−0.0981	−2.4633	−0.4800	−0.5042	1.3586	1.6328	0.3123
0.0060	−0.1887	2.8597	0.9018	1.6500	2.0451	…	1.3160	−1.5767	0.3761	−0.4777	0.3608	1.5960	0.5878

Source: independently developed by the authors with Statistica 10 and Statistica Portable.

**Table 19:** Integral indicators from the social group for Austria, Belgium, Germany, Denmark, Finland, France, the United Kingdom, the Netherlands, Norway, and Sweden for each year from 2000 to 2020.

S	2000	2001	2002	2003	2004	2005	2006	2007	2008
Austria	0.520339	0.669658	0.582071	0.621824	0.646725	0.691718	0.539913	0.623607	0.572861
Belgium	0.467459	0.689518	0.464907	0.550725	0.539356	0.555027	0.625818	0.608212	0.682389
Germany	0.687858	0.999473	1.007299	0.957541	0.847832	0.812678	0.661858	0.741109	0.720046
Denmark	0.3724	0.774359	0.687986	0.704927	0.580935	0.527703	0.35041	0.381299	0.420924
Finland	1.242509	0.326479	0.295786	0.220885	0.135547	0.308065	0.513933	0.462146	0.386133
France	0.487521	1.017774	0.965392	0.831847	0.843371	0.745625	0.740701	0.492299	0.444702
United Kingdom	0.579703	0.817572	0.80146	0.564369	0.734494	0.806294	0.784858	0.782094	0.71258
The Netherlands	0.575295	0.725882	0.705361	0.653182	0.590138	0.412457	0.381644	0.413092	0.434966
Norway	0.792615	0.355713	0.413268	0.358588	0.294789	0.287125	0.171448	0.422023	0.46753
Sweden	0.443125	0.386118	0.309886	0.126218	0.336187	0.31682	0.420964	0.525164	0.539532
Arithmetic mean by country	0.616882	0.676254	0.623342	0.559011	0.554937	0.546351	0.519155	0.545105	0.538166
S	2009	2010	2011	2012	2013	2014	2015	2016	2017
Austria	0.45901	0.451177	0.395522	0.225831	0.092761	0.209443	0.351899	0.339654	0.269981
Belgium	0.567266	0.533025	0.458704	0.416663	0.340523	0.250621	0.190918	0.188889	0.279913
Germany	0.656151	0.55602	0.576929	0.448314	0.405245	0.32524	0.254278	0.250673	0.254315
Denmark	0.334941	0.210746	0.231249	0.29105	0.356107	0.344376	0.404993	0.431818	0.438698
Finland	0.498456	0.533938	0.499799	0.535715	0.538258	0.701405	0.724934	1.047504	1.176602
France	0.477144	0.482063	0.488233	0.430962	0.307406	0.41739	0.271306	0.338435	0.482518
United Kingdom	0.90936	0.989259	1.02402	0.935924	0.810837	0.791908	0.711092	0.692929	0.568481
The Netherlands	0.330197	0.283541	0.323203	0.352177	0.424964	0.407645	0.380463	0.33759	0.43357
Norway	0.729323	0.746778	0.823717	0.99198	0.921307	0.892629	0.880197	0.761039	1.038478
Sweden	0.525658	0.741546	0.595052	0.52055	0.387256	0.573082	0.724287	0.845725	0.926147
Arithmetic mean by country	0.54875	0.552809	0.541643	0.514916	0.458467	0.491374	0.489437	0.523426	0.58687
S	2018		2019		2020		Arithmetic mean by year		
Austria	0.333733		0.30732		0.46649		0.446263658		
Belgium	0.357214		0.347895		0.417202		0.453916154		
Germany	0.280089		0.397073		0.708056		0.597527517		
Denmark	0.219952		0.207395		0.442639		0.414995501		
Finland	1.267368		1.364749		1.476064		0.678870264		
France	0.467873		0.439432		0.365839		0.549420638		
United Kingdom	0.412373		0.487819		0.614381		0.739609813		
The Netherlands	0.477076		0.573958		0.579846		0.466488035		
Norway	1.323611		1.571756		1.680063		0.758284622		
Sweden	0.864932		0.795537		0.633404		0.549389942		
Arithmetic mean by country	0.600422		0.649294		0.738398		0.565476614		

Source: independently developed by the authors.

**Table 20:** Integral indicators from the demographic group for Austria, Belgium, Germany, Denmark, Finland, France, the United Kingdom, the Netherlands, Norway, and Sweden for each year from 2000 to 2020.

D	2000	2001	2002	2003	2004	2005	2006	2007	2008
Austria	0.221459	1.069901	0.911991	0.855094	0.873164	0.478948	0.70071	0.584384	0.593539
Belgium	0.479692	0.556026	0.51545	0.457372	0.407774	0.432104	0.268882	0.330566	0.318181
Germany	0.477104	1.608672	1.418087	1.239279	0.830234	0.79156	0.862039	0.783571	0.752204
Denmark	0.849044	0.644273	0.667455	0.689518	0.692231	0.692951	0.669466	0.638714	0.604489
Finland	0.814519	0.98651	0.958705	0.899002	0.810967	0.778715	0.646033	0.597672	0.430084
France	1.130187	0.283587	0.296497	0.408893	0.396474	0.455886	0.328591	0.323981	0.304791
United Kingdom	0.214154	0.470687	0.60023	0.715818	0.765293	0.760613	0.770517	0.753044	0.691369
The Netherlands	0.508192	0.585146	0.607151	0.453998	0.583983	0.745412	0.624502	0.53098	0.544757
Norway	0.417306	0.625276	0.538738	0.357208	0.396639	0.434399	0.362917	0.419346	0.362366
Sweden	1.08143	0.280212	0.266909	0.247281	0.225685	0.119111	0.153229	0.137723	0.197711
Arithmetic mean by country	0.619309	0.711029	0.678121	0.632346	0.598244	0.56897	0.538689	0.509998	0.479949
D	2009	2010	2011	2012	2013	2014	2015	2016	2017
Austria	0.50868	0.472273	0.521527	0.533259	0.598403	0.651676	0.570285	0.585128	0.513148
Belgium	0.27342	0.22552	0.1672	0.180247	0.091957	0.289102	0.268085	0.406765	0.368451
Germany	0.698316	0.62143	0.434339	0.34085	0.213817	0.428875	0.293443	0.25272	0.211845
Denmark	0.542909	0.406387	0.367	0.318833	0.421991	0.527073	0.625561	0.655129	0.617364
Finland	0.483104	0.446281	0.270831	0.249375	0.361616	0.31996	0.551089	0.607458	0.677355
France	0.297839	0.286004	0.343298	0.407818	0.557093	0.750441	0.86274	0.922899	0.979198
United Kingdom	0.450743	0.492555	0.283595	0.192923	0.261307	0.207312	0.25909	0.237717	0.3232
The Netherlands	0.551282	0.540977	0.370941	0.365453	0.365062	0.275252	0.149303	0.37389	0.488037
Norway	0.461257	0.499447	0.534025	0.624272	0.64648	0.666011	0.655275	0.604694	0.552049
Sweden	0.290666	0.156872	0.449866	0.557083	0.665834	0.710169	0.692066	0.942906	1.00301
Arithmetic mean by country	0.455822	0.414775	0.374262	0.377011	0.418356	0.482587	0.492694	0.558931	0.573366
D	2018		2019		2020		Arithmetic mean by year		
Austria	0.415883		0.315223		0.221459		0.580768335		
Belgium	0.514261		0.678158		0.479692		0.367090594		
Germany	0.274411		0.482015		0.477104		0.64247201		
Denmark	0.658844		0.811892		0.849044		0.616674818		
Finland	0.667964		0.643381		0.820271		0.620042404		
France	0.940008		1.168709		1.130187		0.598815426		
United Kingdom	0.343949		0.311263		0.214154		0.443787213		
The Netherlands	0.547762		0.587723		0.508192		0.490856852		
Norway	0.490456		0.310531		0.417306		0.4940951		
Sweden	1.051391		1.146781		1.08143		0.545588914		
Arithmetic mean by country	0.590493		0.645568		0.619884		0.540019167		

Source: independently developed by the authors.

**Table 21:** Integral indicators for the economic group for Austria, Belgium, Germany, Denmark, Finland, France, the United Kingdom, the Netherlands, Norway, and Sweden for each year from 2000 to 2020.

E	2000	2001	2002	2003	2004	2005	2006	2007	2008
Austria	0.641588	0.64328	0.635571	0.599529	0.539003	0.384616	0.361684	0.239181	0.308264
Belgium	0.760946	0.730126	0.75542	0.714677	0.699519	0.56709	0.334923	0.251568	0.212771
Germany	0.697014	0.689297	0.5228	0.617901	0.455707	0.336166	0.487723	0.375297	0.387642
Denmark	1.269232	1.172255	0.531697	0.761613	0.328192	0.41135	0.578707	0.755736	0.960982
Finland	0.619544	0.729229	0.505608	0.643939	0.57535	0.395409	0.441038	0.386264	0.577412
France	0.988559	0.926943	0.900367	0.711222	0.730736	0.715239	0.671354	0.537625	0.35113
United Kingdom	1.353265	1.360284	1.186417	1.085414	0.922396	0.698346	0.538335	0.539117	0.484249
The Netherlands	1.061647	1.019415	0.999025	0.85368	0.712219	0.604726	0.458208	0.630736	0.74824
Norway	0.527728	0.74535	0.737574	0.598796	0.946353	1.075097	0.946464	1.151815	1.104673
Sweden	1.192891	1.054795	1.084724	0.850921	0.545317	0.287919	0.217349	0.392802	0.539382
Arithmetic mean by country	0.911241	0.907097	0.78592	0.743769	0.645479	0.547596	0.503579	0.526014	0.567474
E	2009	2010	2011	2012	2013	2014	2015	2016	2017
Austria	0.274123	0.384839	0.541832	0.442838	0.519201	0.546534	0.357353	0.306454	0.425051
Belgium	0.109825	0.204078	0.200738	0.202667	0.161855	0.202958	0.232891	0.348158	0.383838
Germany	0.50863	0.473747	0.204153	0.326432	0.17535	0.294897	0.504879	0.571075	0.498242
Denmark	1.132742	1.228054	1.261449	0.895134	1.000007	0.917767	0.783718	0.811825	0.762715
Finland	0.27852	0.288457	0.468009	0.386083	0.451877	0.486807	0.293446	0.401405	0.241077
France	0.372582	0.324987	0.342111	0.468392	0.436657	0.428681	0.518265	0.566044	0.636517
United Kingdom	0.741724	0.663785	0.592047	0.463068	0.448212	0.342552	0.349716	0.345377	0.512967
The Netherlands	0.630906	0.654537	0.626224	0.5897	0.56089	0.561581	0.415227	0.15222	0.189128
Norway	1.498699	1.205125	1.09275	1.343317	1.342156	1.446531	0.76946	0.982194	0.95607
Sweden	0.315485	0.525488	0.50671	0.894329	1.017146	0.969335	0.774771	0.746636	0.748064
Arithmetic mean by country	0.586324	0.59531	0.583602	0.601196	0.611335	0.619764	0.499973	0.523139	0.535367
E	2018		2019		2020		Arithmetic mean by year		
Austria	0.480556		0.527024		0.41619		0.45593865		
Belgium	0.255987		0.253042		0.37729		0.379065073		
Germany	0.377946		0.316985		0.362035		0.437329551		
Denmark	0.733253		0.722606		0.71024		0.844251193		
Finland	0.331327		0.365635		0.417927		0.442112586		
France	0.559482		0.556872		0.533233		0.584618905		
United Kingdom	0.522843		0.541029		0.671447		0.683932719		
The Netherlands	0.487058		0.606656		0.542678		0.624033412		
Norway	1.018847		0.929519		0.793775		1.010109137		
Sweden	0.836433		0.785995		0.777173		0.717317373		
Arithmetic mean by country	0.560373		0.560536		0.560199		0.61787086		

Source: independently developed by the authors.

**Table 22:** Integral indicators for the behavioral group for Austria, Belgium, Germany, Denmark, Finland, France, the United Kingdom, the Netherlands, Norway, and Sweden for each year from 2000 to 2020.

B	2000	2001	2002	2003	2004	2005	2006	2007	2008
Austria	0.76348	1.490589	1.544248	1.493704	1.524242	1.348795	1.199451	1.050159	0.774533
Belgium	0.663904	0.820514	0.570088	0.566728	0.555421	0.674704	0.434076	0.392326	0.42379
Germany	0.621074	0.691548	0.692569	0.788565	0.720487	0.69507	0.669492	0.65492	0.624317
Denmark	0.461076	0.61188	1.008464	0.907241	0.466401	0.643967	0.63783	0.923116	0.975779
Finland	0.83002	0.91604	0.895853	0.927867	0.890512	0.793061	0.747151	0.8501	0.755944
France	1.308481	1.1939	1.13169	1.104289	0.85662	0.877558	0.802386	0.60932	0.661087
United Kingdom	0.374232	0.89636	0.952175	0.996043	0.880568	1.276126	0.789962	0.754979	1.040462
The Netherlands	0.295815	0.525132	0.534258	0.628757	0.589846	0.446049	0.463609	0.460062	0.42427
Norway	0.763421	0.691366	0.486418	0.526787	0.353284	0.247994	0.148048	0.202931	0.159672
Sweden	0.756483	0.575197	0.636727	0.585284	0.353516	0.361253	0.558	0.627176	0.697489
Arithmetic mean by country	0.683799	0.841253	0.845249	0.852526	0.71909	0.736458	0.645	0.652509	0.653734
B	2009	2010	2011	2012	2013	2014	2015	2016	2017
Austria	1.003705	0.700909	0.902782	0.671211	0.286652	0.775416	0.640113	0.883558	0.846685
Belgium	0.486976	0.449938	0.317249	0.579881	0.494292	0.558832	0.711258	0.752829	0.73324
Germany	0.60873	0.579486	0.291647	0.211876	0.353123	0.628361	0.457176	0.597628	0.595737
Denmark	1.025432	0.905029	0.737282	0.420927	0.413471	0.359445	0.389816	0.394066	0.688966
Finland	0.677778	0.630213	0.298409	0.280693	0.429226	0.510226	0.501626	0.503612	0.664885
France	0.639567	0.685384	0.724204	0.597541	0.676193	0.600046	0.528016	0.741414	0.727614
United Kingdom	0.837396	0.490496	0.473452	0.337275	0.251645	0.277845	0.249329	0.338803	0.349666
The Netherlands	0.383847	0.576982	0.778841	0.541671	0.585417	0.569404	0.372656	0.343068	0.363897
Norway	0.217648	0.304753	0.410169	0.438767	0.337772	0.524715	0.404996	0.614459	0.633677
Sweden	0.778152	0.798033	0.801286	0.82361	0.880095	0.800559	0.956619	0.936614	0.833921
Arithmetic mean by country	0.665923	0.612122	0.573532	0.490345	0.470789	0.560485	0.52116	0.610605	0.643829
	2018		2019		2020		Arithmetic mean by year		
Austria	0.824505		0.834128		0.720501		0.965684141		
Belgium	0.735235		0.768579		0.495618		0.580260849		
Germany	0.56557		0.651845		0.574712		0.584472887		
Denmark	0.54363		0.669148		0.579617		0.655361146		
Finland	0.708465		0.749043		0.776143		0.682707958		
France	0.721263		0.710613		0.62248		0.78665076		
United Kingdom	0.35072		0.511196		0.457011		0.61360673		
The Netherlands	0.43896		0.485259		0.434853		0.487745434		
Norway	0.64806		0.78172		0.798765		0.461686888		
Sweden	0.850623		0.963997		0.843265		0.734185598		
Arithmetic mean by country	0.638703		0.712553		0.630296		0.655236239		

Source: independently developed by the authors.

**Table 23:** Integral indicators of healthcare system development in Austria, Belgium, Germany, Denmark, Finland, France, the United Kingdom, the Netherlands, Norway, and Sweden for each year from 2000 to 2020.

R	2000	2001	2002	2003	2004	2005	2006	2007	2008
Austria	0.487425	0.910413	0.849595	0.83069	0.825306	0.643869	0.636493	0.550045	0.533784
Belgium	0.580151	0.692279	0.566788	0.565161	0.540666	0.550385	0.395485	0.375322	0.374061
Germany	0.613938	0.935657	0.848037	0.872008	0.693355	0.622653	0.656981	0.614654	0.601704
Denmark	0.655864	0.773438	0.70442	0.761266	0.4981	0.557881	0.542459	0.642022	0.698899
Finland	0.849357	0.681057	0.598657	0.5869	0.487154	0.523713	0.575124	0.548781	0.518879
France	0.918816	0.751778	0.734882	0.718928	0.676387	0.679635	0.601739	0.4781	0.421162
United Kingdom	0.500742	0.827642	0.858595	0.812942	0.822004	0.859816	0.712128	0.69972	0.705845
The Netherlands	0.550465	0.690538	0.691447	0.631635	0.616846	0.536635	0.474353	0.502281	0.523703
Norway	0.604189	0.581848	0.531626	0.44834	0.444653	0.427034	0.305571	0.450983	0.415772
Sweden	0.810926	0.506172	0.488888	0.353095	0.347765	0.2503	0.297402	0.365356	0.44758
Arithmetic mean by country	0.657187	0.735082	0.687294	0.658097	0.595223	0.565192	0.519773	0.522726	0.524139
R	2009	2010	2011	2012	2013	2014	2015	2016	2017
Austria	0.503448	0.489632	0.563604	0.434967	0.301483	0.490413	0.462877	0.48164	0.472536
Belgium	0.301791	0.324131	0.264362	0.306509	0.223723	0.301085	0.303441	0.376709	0.412761
Germany	0.613721	0.554968	0.349495	0.320631	0.270644	0.400962	0.362264	0.383458	0.355608
Denmark	0.677927	0.55545	0.530045	0.432421	0.499267	0.494672	0.527453	0.548485	0.61421
Finland	0.461746	0.456212	0.3708	0.346877	0.440795	0.485899	0.492444	0.598877	0.597843
France	0.428978	0.418619	0.451421	0.470945	0.474202	0.532776	0.503077	0.601707	0.683949
United Kingdom	0.710329	0.631113	0.534146	0.409792	0.393178	0.353555	0.356013	0.372604	0.426072
The Netherlands	0.458213	0.490595	0.491745	0.450287	0.475079	0.435223	0.306192	0.284935	0.347386
Norway	0.57555	0.608366	0.666355	0.777271	0.720853	0.819598	0.651119	0.725952	0.767685
Sweden	0.440084	0.469966	0.574178	0.67983	0.693137	0.749655	0.780715	0.864155	0.872494
Arithmetic mean by country	0.517179	0.499905	0.479615	0.462953	0.449236	0.506384	0.47456	0.523852	0.555054
R	2018		2019		2020		Arithmetic mean by year		
Austria	0.484258		0.454274		0.419532		0.563156461		
Belgium	0.43121		0.462823		0.439828		0.418508155		
Germany	0.358017		0.445942		0.514897		0.542361624		
Denmark	0.490249		0.534171		0.627165		0.588850658		
Finland	0.667664		0.700275		0.791637		0.560985324		
France	0.64906		0.671424		0.608654		0.59410664		
United Kingdom	0.401586		0.452687		0.448256		0.585179249		
The Netherlands	0.486179		0.561361		0.513519		0.500886523		
Norway	0.809135		0.771703		0.816534		0.615244616		
Sweden	0.896868		0.91182		0.81854		0.600901282		
Arithmetic mean by country	0.567423		0.596648		0.599856		0.557018053		

Source: independently developed by the authors.

## Data Availability

Not applicable.
